# 2019 Novel Coronavirus-Infected Pneumonia on CT: A Feasibility Study of Few-Shot Learning for Computerized Diagnosis of Emergency Diseases

**DOI:** 10.1109/ACCESS.2020.3033069

**Published:** 2020-10-22

**Authors:** Yaoming Lai, Guangming Li, Dongmei Wu, Wanmin Lian, Cheng Li, Junzhang Tian, Xiaofen Ma, Hui Chen, Wen Xu, Jun Wei, Yaqin Zhang, Guihua Jiang

**Affiliations:** Guangzhou Perception Vision Medical Technology Inc. Guangzhou 510000 China; Xiangyang Central HospitalAffiliated Hospital of Hubei University of Arts and Science Xiangyang 441000 China; Nanxishan Hospital of Guangxi Zhuang Autonomous Region 477248 Guilin 541000 China; Department of Medical ImagingGuangdong Second Provincial General Hospital 485285 Guangzhou 510000 China; Department of RadiologyThe Fifth Affiliated HospitalSun Yat-sen University 26469 Zhuhai 519000 China

**Keywords:** COVID-19, chest CT, 2019 novel coronavirus-infected pneumonia, few-shot learning

## Abstract

COVID-19 is an emerging disease with transmissibility and severity. So far, there are no effective therapeutic drugs or vaccines for COVID-19. The most serious complication of COVID-19 is a type of pneumonia called 2019 novel coronavirus-infected pneumonia (NCIP) with about 4.3% mortality rate. Comparing to chest Digital Radiography (DR), it is recently reported that chest Computed Tomography (CT) is more useful to serve as the early screening and diagnosis tool for NCIP. In this study, aimed to help physicians make the diagnostic decision, we develop a machine learning (ML) approach for automated diagnosis of NCIP on chest CT. Different from most ML approaches which often require training on thousands or millions of samples, we design a few-shot learning approach, in which we combine few-shot learning with weakly supervised model training, for computerized NCIP diagnosis. A total of 824 patients are retrospectively collected from two Hospitals with IRB approval. We first use 9 patients with clinically confirmed NCIP and 20 patients without known lung diseases for training a location detector which is a multitask deep convolutional neural network (DCNN) designed to output a probability of NCIP and the segmentation of targeted lesion area. An experienced radiologist manually localizes the potential locations of NCIPs on chest CTs of 9 COVID-19 patients and interactively segments the area of the NCIP lesions as the reference standard. Then, the multitask DCNN is furtherly fine-tuned by a weakly supervised learning scheme with 291 case-level labeled samples without lesion labels. A test set of 293 patients is independently collected for evaluation. With our NCIP-Net, the test AUC is 0.91. Our system has potential to serve as the NCIP screening and diagnosis tools for the fight of COVID-19’s endemic and pandemic.

## I. Introduction

On December 31, 2019, the cases of pneumonia unknown etiology in Wuhan are firstly reported to WHO China Country Office and subsequently named as COVID-19. Since then, its pathogen has been confirmed by multiple studies [1], [2] and more than 100,000 confirmed cases have been reported globally. Its pandemic has impacted the global economy as well as normal lifestyle in certain countries especially China. Researchers have been working tirelessly to develop therapeutic drugs or vaccines for the treatment and prevention of COVID-19. However, there is still a long way for any effective medicine available for public use. The only effective method in clinical practice is to detect and diagnose COVID-19 early to prevent its further spread.

The fact that asymptomatic persons are potential sources of COVID-19 infection [3], [4] makes the early diagnosis of COVID-19 extremely challenging. The population-based screening may be the only way to prevent the community outbreak. Currently, the reverse transcription polymerase chain reaction (RT-PCR) or gene sequencing for respiratory or blood specimens are tools for COVID-19 screening. However, the availabilities of these tools and its lag time are some of the limitations in pandemic setting. In additional, the test kits such as RT-PCR have been found not 100 percent accurate even for the symptomatic patients which may be due to the requirement of skill levels or other unknown reasons. It is also reported in [5]–[8] that the COVID-19 patients have varieties of symptoms which make them harder to be distinguished from other flu viruses.

So far, the complete clinical complication related to COVID-19 is not fully understood. A study suggested that 16% of COVID-19 cases has serious illness. The most serious complication of COVID-19 is a certain type of pneumonia called 2019 novel coronavirus-infected pneumonia (NCIP) with about 4.3% mortality rate. In a recent study of 1014 patients conducted in Wuhan [9], only 59% has positive RT-PCR results instead 88% has positive findings of NCIPs on chest CT. A comparative study [10] on the clinical features of NCIP to other pneumonias suggests that the COVID-19 infection causes similar clinical onsets to other pneumonias. These findings have promoted the use of chest CT as additional screening and diagnostic tool for the clinical triage of COVID-19.

Since then a variety of imaging features have been reported [11]. The question is whether or how the incremental experiences or knowledge of NCIP diagnosis by chest CTs could be transparent to the radiologists with less experiences to diagnose the NCIPs globally. Thus, in this study, we attempt to develop a few-shot learning-based lesions detection and classification approach (NCIP-Net) to aid less experienced radiologist for the detection and diagnosis of NCIPs in the outbreak of COVID-19. The rational of the few-shot learning [12] in weakly supervised manner used in our algorithm design is to keep the ability of the proposed method in learning the incremental information both automatically and continuously to adapt the reality that the updates of knowledge and treatment experiences of NCIPs are fast and ongoing.

## II. Material & Method

### A. Study Material

We retrospectively collected 815 patients with chest CT images and clinical diagnosis results for algorithm development and testing. IRB approval has been obtained from local ethical review system for this study. Our patient cases were sequentially collected from two different sources including 531 patients (187 NCIP cases, 132 other pneumonia cases, 96 benign nodule cases, 96 lung cancer cases, and 20 cases without known lung diseases) from Hospital A, and 293 cases (136 NCIP cases, 36 other pneumonia cases, and 121 cases without known lung diseases) from Hospital B, respectively. Table 1 summarized the detailed information of our data set. The NCIP lesions of 9 COVID-19 patients from hospital A were identified and interactively contoured on all CT slices. The cases from Hospital A were used for development our NCIP-Net, while those from Hospital B were used for independent testing. The processes of training and testing are detailed in Section C.

**TABLE 1:** Information of Our Dataset

Source	Diagnosis	# of cases	Imaging Protocol	
			Vendor	Slice thickness (mm)
Hospital A	NCIP	187	UIH/Philips	0.625 - 2.5
	Other pneumonia	132		
	Lung Nodule/Cancer	96/96	Toshiba/GE	1.0 – 1.25
	Without known lung diseases	20	UIH/Philips	0.625 - 2.5
Hospital B	NCIP	136	UIH/Philips	0.625 – 5
	Other pneumonia	36		
	Without known lung disease	121		

### B. Study Method

**FIGURE 1.**
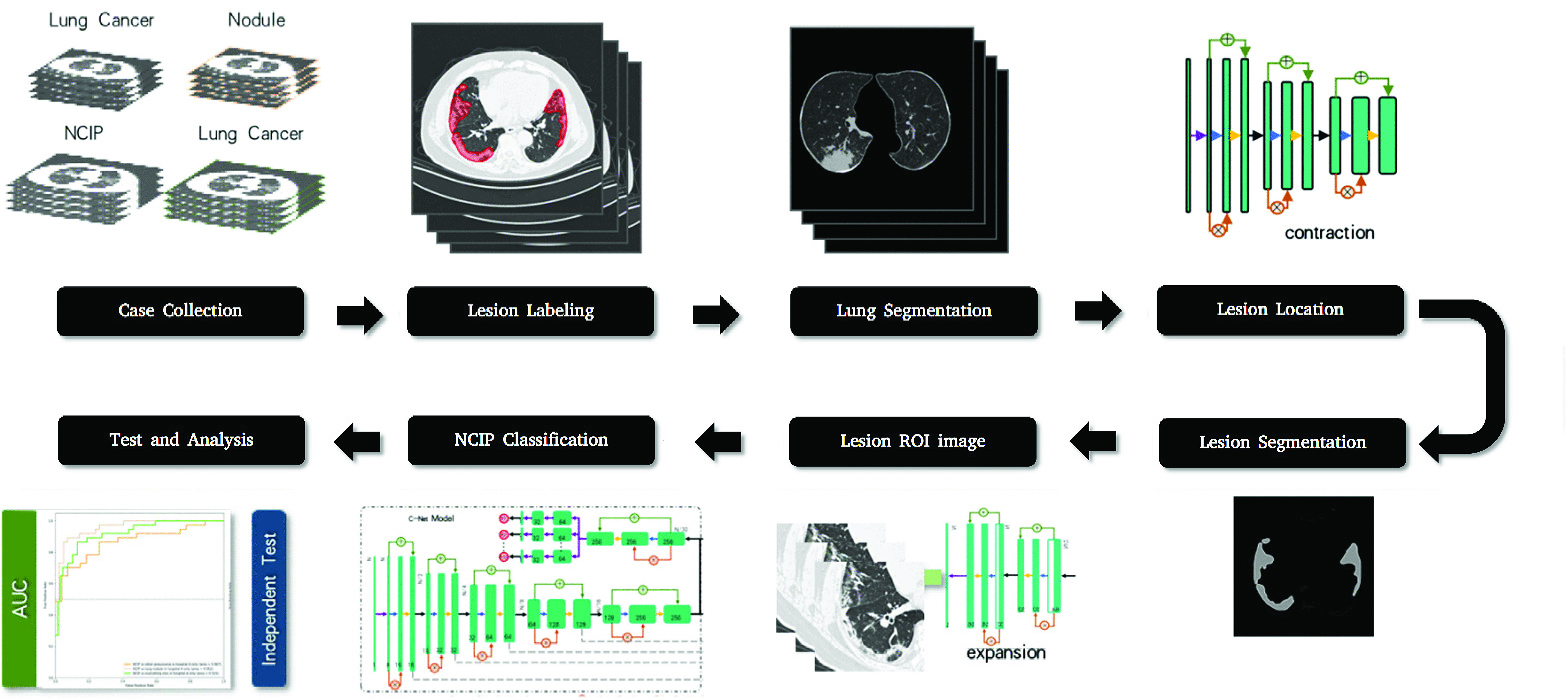
Diagram of machine learning (ML) algorithm for the diagnosis of NCIP.

Figure 1 illustrated the processes of our NCIP-Net. The whole NCIP-Net includes three stages with three subnetworks for lung segmentation, lesions detection and lesions-based disease classification, respectively. To reduce the computation time as well as avoid false positives, a previously proposed multiscale deep convolutional neural network (DCNN) was firstly applied on the input image for lung segmentation. Thus, the following processes are just needed to conduct on the segmented lung regions. For lesions detection and segmentation, only 9 NCIP patients and 20 normal cases were used for a region-of-interest (ROI) based network pretrained with the few-short learning strategy. Afterward, locations of suspicious lesions were proposed as the input of C-Net for the fine turning of entire NCIP-Net.

Stage 1: Lung Segmentation

For each input CT volume, we firstly segmented the lung area using our previously developed multiscale lung segmentation method [13], based on deep convolutional neural network (DCNN) trained by multiscale Dice (MD) loss. The purpose of this process was to limit the lesion detection in a subregion to reduce the computation time as well as avoid false positives (FPs).

Stage 2: Lesion Detection

A multitask DCNN was constructed for the NCIP lesion detection, segmentation and prediction of COVID-19 probability. Our multitask DCNN was based on a U-Net [14], which included encoding and decoding phases, and trained by a two-stage training strategy: 1) the trainings of lesion segmentation and the image-based NCIP probability; and 2) the trainings of lesion-based NCIP probability and the case-based prediction of COVID-19 probability.

Stage 3: Disease Classification

**FIGURE 2.**
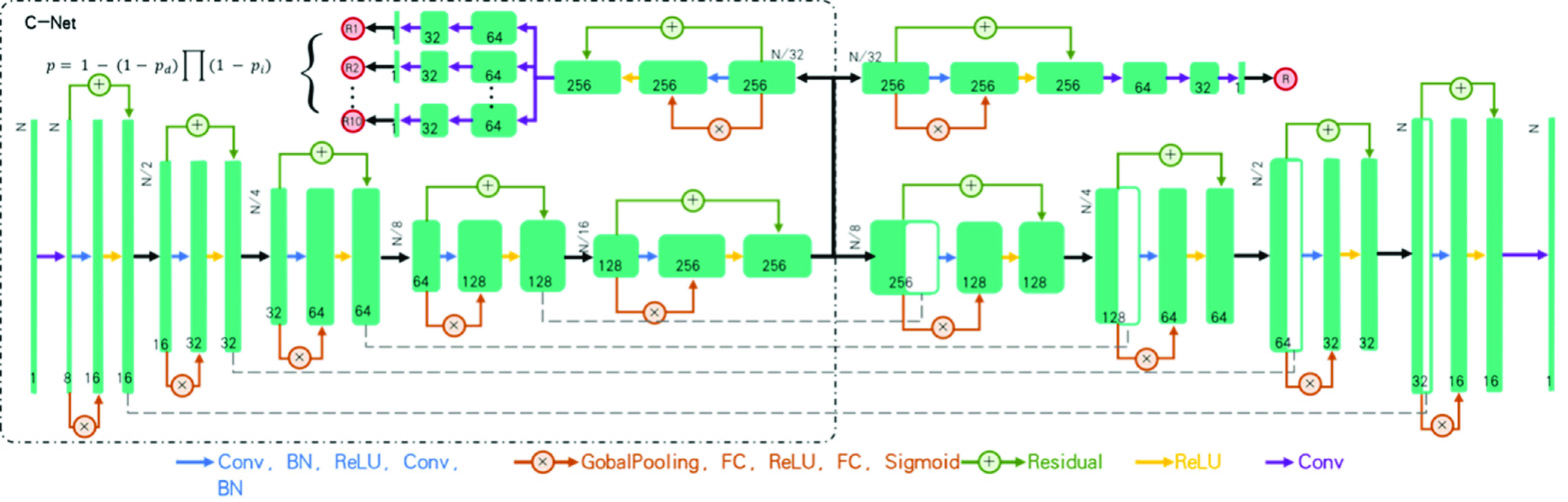
Deep convolutional neural network (DCNN) architecture for the machine diagnosis of NCIPs. The training of this DCNN is a two-stage processes. In the first stage, we train the DCNN to distinguish the region of interests (ROIs) with the likelihood of NCIP into two classes: NCIPs or otherwise. In the second stage, we fine-tune the DCNN to predict the case-based probability of COVID-19 by ensembling the most significant location proposals self-produced in the DCNN.

Figure 2 illustrated the DCNN architecture used in this study. In the first training stage, the DCNN was trained with region of interests (ROIs). At the end of the encoding phase in multitask DCNN detector, the output of encoding was bifurcated into two branches: one for decoding phase used for lesion segmentation and the other for the likelihood prediction of NCIP lesion. In the branch of likelihood prediction, a prediction unit consisted of a SE-residual block, three convolutional layers, an average pooling layer and a sigmoid function were used to generate the likelihood score of NCIP for ROI. The cross-entropy loss was used for the task of lesion-based likelihood training while a weighted loss containing cross-entropy and Dice losses was used for the task of lesion segmentation.

**FIGURE 3.**
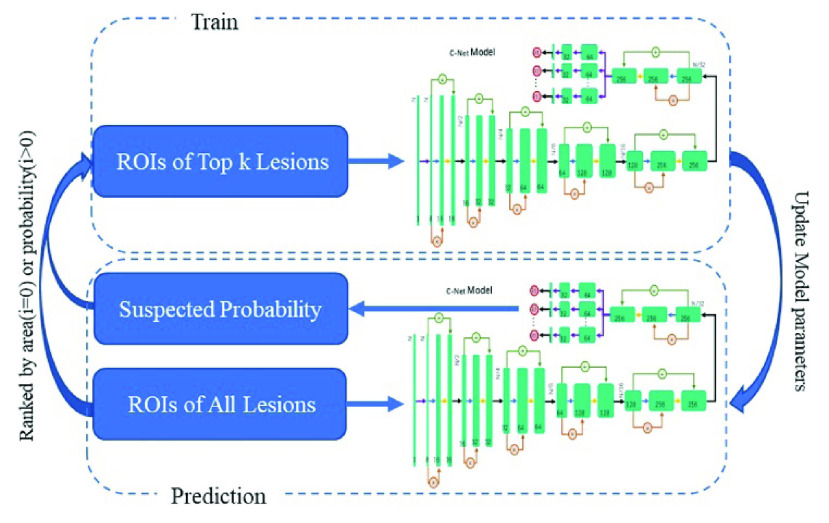
Diagram of the mechanism for candidate proposal. In the initial stage, a maximum of 10 NCIP candidate proposals were selected based on the ranked weighted area (from largest to smallest) resulted from target segmentation processes. The selected NCIP candidates were used to train the C-Net iteratively. During the training, the candidate proposals were randomly selected from the first 50 proposals at each iteration.

In the second training stage, the case-based prediction using CT volume patches as the input, denoted as C-Net model hereon, was trained to identify a maximum of 10 proposals with the likelihood of NCIP lesion to output the probability of COVID-19 of the case. The mechanism of candidate proposals is shown in the Figure 3. Assuming the independent contribution of each location proposal, the case-based probability was calculated as,

\begin{equation*} P=1-(1-P_{d})\Pi (1-P_{i})\tag{1}\end{equation*}

where P was the case-based probability; $P_{i}$ was the probability of ith location proposal; and $P_{d}$ was a trainable parameter to serve as the regularization factor during the training.

### C. Training Design

Due to the resource constrains, the lesions of NCIPs were manually identified and interactively segmented on every CT slices of 9 COVID-19 patients collected from Hospital A, a total of 2178 suspicious NCIP lesions on 1345 slices were segmented as the reference standard by an experienced radiologist. These individual NCIP lesions were used for ROI-based training of DCNN in the first training stage detailed in the section B. 20 normal cases from Hospital A were also used in the first stage to balance the positive and negative classes, 2178 locations from 1345 slices were randomly generated from 20 normal cases. During the training, auto data augmentation [15] was employed for synthetic data generation. The number of epochs (100 in this study) was used as the stopping criterion to terminate the training of the first stage.

**FIGURE 4.**
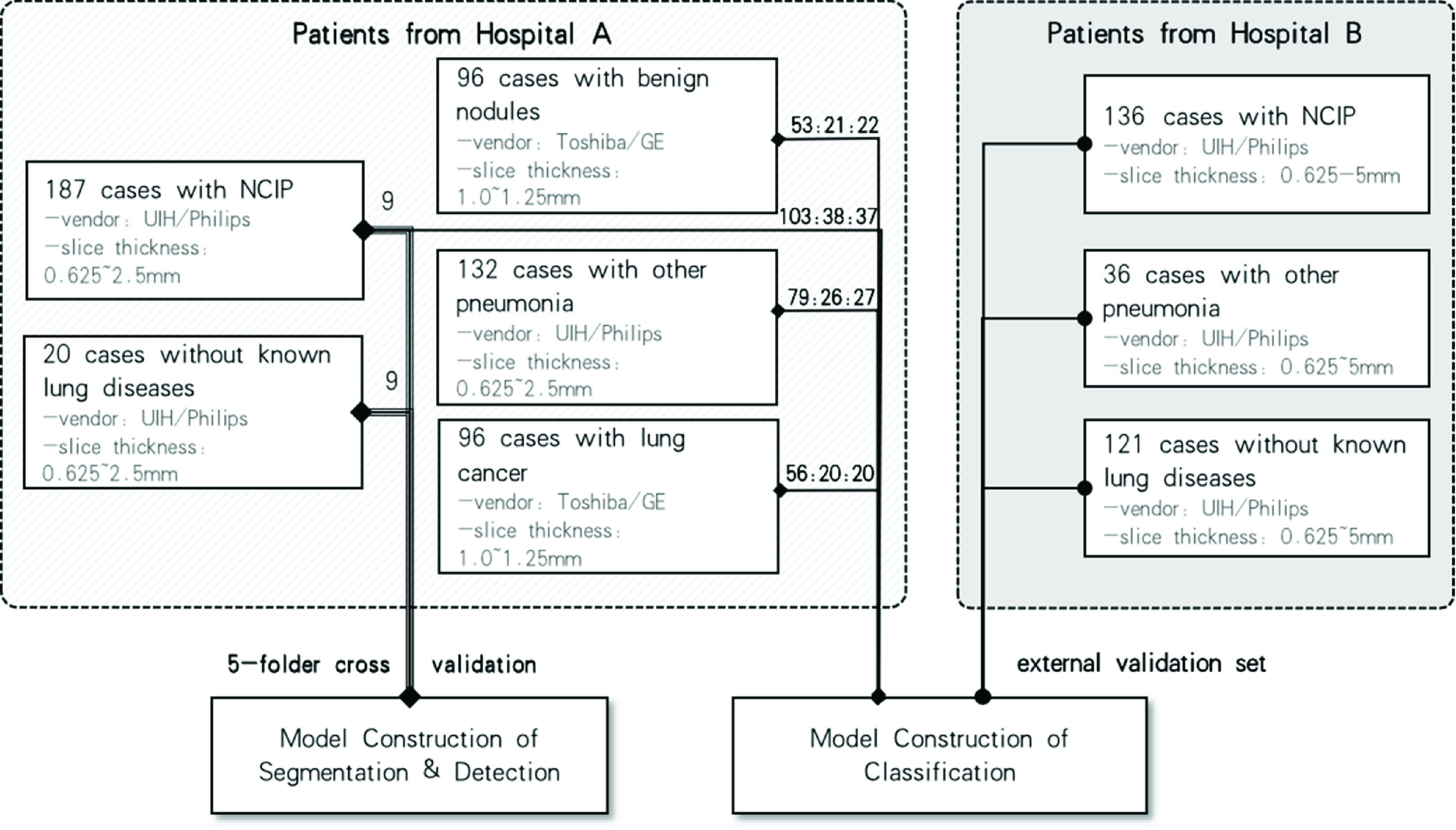
Diagram of NCIP-Net training, validation, and testing.

During the second stage, we randomly divided the remaining 502 cases from Hospital A into three sets: the training set with 291 cases (103 of NCPs, 79 of other pneumonia, and 109 of lung nodules), the validation set with 105 cases (38 of NCIPs, 26 of other pneumonia, and 41 of lung nodules) and test set with 106 cases (37 of NCIPs, 27 of other pneumonia, and 41 of lung nodules). Finally, 293 patients of hospital B were served as the external validation set to evaluate our NCIP-Net. The area under the receiver operating characteristic (ROC) curve was serviced as the figure-of-merit of our study. A diagram of the training, validation, and testing processes was illustrated in Figure 4.

## III. Experiment Result

As a compared experiment to NCIP-Net, we used the Mask-R-CNN [16] as a segmentation network to extract the lesion zone and then trained a SVM to predict the likelihood of the COVID-19 based on the segmentation results (we use Mask-R-CNN-SVM for short in the following paragraph). we used the same preprocess as NCIP-Net for Mask-R-CNN during training.

**FIGURE 5.**
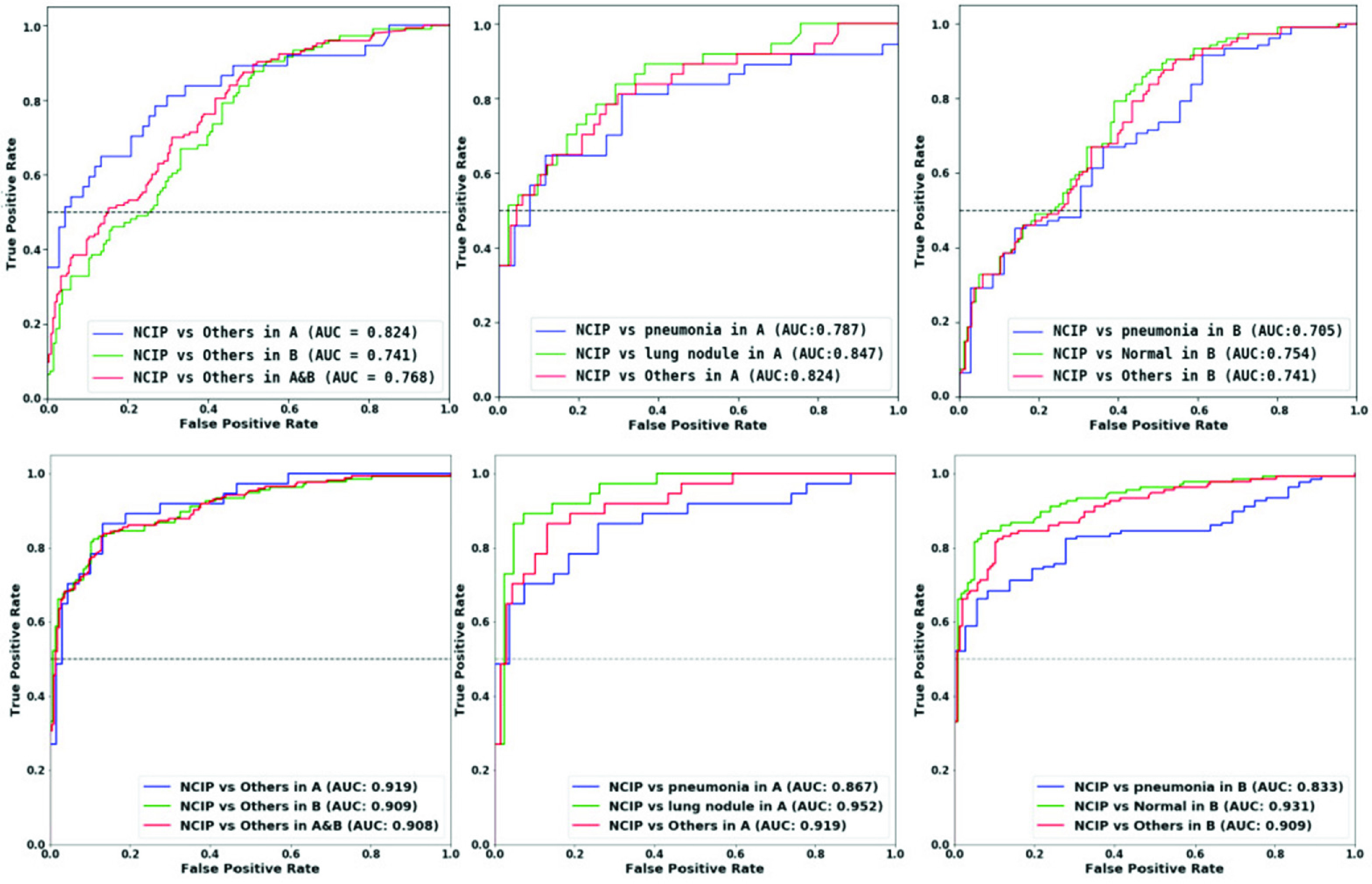
The receiver operating characteristic (ROC) curves for the case-based performances of COVID-19 DIAGNOSIS. The first row is the Mask-R-CNN-SVM ROC CURVES. The second row is the NCIP-Net ROC curve. Left: Three ROC Curves show the test result with 106 cases (37 OF NCIPS, 27 of other pneumonia, and 42 of lung nodules), the external validation result with 293 cases (136 NCIP cases, 36 other pneumonia cases, and 121 normal cases) from hospital B, and the superset including validation and test sets. Middle: Three ROC curves show the validation results including NCIPS vs Other two patient groups. Right: Three ROC curves show the testing results including ncips vs other two patient groups.

Figure 5 illustrated the case-based performances of COVID-19 diagnosis in different data sets in terms of the ROC analysis of NCIP-Net and Mask-R-CNN-SVM. The second row show the ROC curve of NCIP-Net. The test, external validation, and test & external validation AUCs of NCIP-Net were 0.919, 0.908 and 0.908 compared to 0.824, 0.741, 0.761 in Mask-R-CNN-SVM respectively. There were no significant differences between three pair-wised ROC analyses (p>0.05) in NCIP-Net. In test, NCIPs vs all others, NCIPs vs lung nodule cases, and NCIP vs other pneumonia cases of NCIP-Net were 0.919, 0.952 and 0.867 compared to 0.824, 0.847, 0.787 in Mask-R-CNN-SVM respectively. Significant difference was observed between the curve of NCIPs vs lung nodule cases and the curve of NCIPs vs other pneumonia cases (p<0.05) in NCIP-Net. Finally, in external validation, the AUCs of NCIPs vs all others, NCIPs vs normal cases, and NCIP vs other pneumonia cases of NCIP-Net were 0.909, 0.931, and 0.833 compared to 0.741, 0.754, 0.705 in Mask-R-CNN-SVM, respectively. It was significant different between the curve of NCIPs vs normal cases and the curve of NCIPs vs other pneumonia cases (p<0.05) in NCIP-Net.

As showed in Table 2 above, the overall accuracies of NCIPs vs other pneumonia cases in test and external validation sets of NCIP-Net were 0.757 and 0.744 while those of NCIPs vs lung nodule cases in test set and NCIPs vs normal cases in external validation set were 0.861 and 0.829, compared to 0.698, 0.599, 0.744 and 0.626 in Mask-R-CNN-SVM respectively.

**TABLE 2:** The Accuracy, Sensitivity, Specificity of Case-Based COVID-19 Diagnosis of Our Method Compared to Mask-R-CNN -SVM Method

Method	Source	Image Sets	Accuracy	Sensitivity	Specificity
Mask-R-CNN-SVM	Test	vs Lung Nodules	0.744	0.649	0.829
		vs Other Pneumonia	0.698	0.649	0.769
	External Validation	vs Other Pneumonia	0.599	0.575	0.667
		vs Without Known Lung Diseases	0.626	0.646	0.719
NCIP-Net	Test	vs Lung Nodules	0.861	0.757	0.952
		Vs Other Pneumonia	0.757	0.757	0.815
	External Validation	vs Other Pneumonia	0.744	0.713	0.861
		vs Without Known Lung Diseases	0.829	0.713	0.959

note that we used the case-based probability of 0.5 as the thresholding value to generate all of the results in this table.

It is apparently that NCIP-Net is outperformance the Mask-R-CNN-SVM at every aspect of all datasets, especially in the external validation.

**FIGURE 6.**
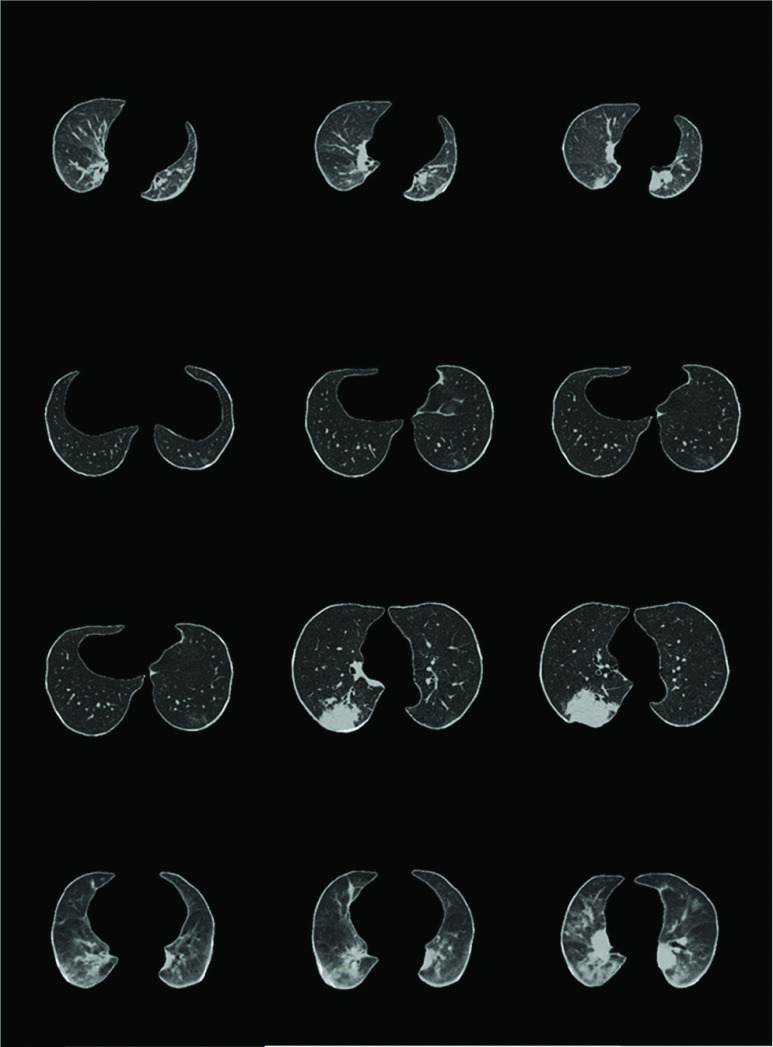
Image examples of false negative and false positive cases in different patient groups. From top to bottom (computerized probability): a 61-year-old male with NCIP; a 17-year-old female with NCIP; a 59-year-old male with lung cancer; and an 88-year-old male with other pneumonia.

Figure 6 showed the matrix of image examples where CT slices in false positive cases and false negative cases were presented in each patient group.

## IV. Discussion

In this study, we built a computerized NCIPs diagnosis system based on deep learning method. Our purpose was to exam whether machine learning could be useful in early diagnosis of NCIPs caused by COVID-19. Since this disease is new, the knowledge about its appearances on CT images is keeping updating. We chose an DCNN approach over feature engineering. The advantage of DCNN is that it could generate the characteristic map automatically based on the training samples. However, one of the drawbacks in DCNN is that it often required a relatively large training samples to avoid over training. As obviously showed in the compared experiment above, the limited dataset leads to over training of the feature extract, furtherly, effects the generation ability of network in another data set especially the external data set. Due to the limitation of available COVID-19 cases, few-shot learning and weakly supervised training are used in our work to train a multi-task DCNN which is to learn a correlated task of NCIP lesion detection, lesion segmentation, and case-based likelihood assessment of COVID-19. The results show that NCIP-Net has better generation ability and the potential of our DCNN in the future clinical practice in aiding the radiologist to make the early decision of COVID-19 diagnosis.

Recently, deep learning approach has been widely used in different areas. In general, deep learning requires a large and well-defined data source before its model construction and training. In reality, however, it is often difficult and very expensive to obtain such data sets for building the ideal models especially in health care setting. One-shot or few-shot learning aims to learn information about the abstraction of targets from one or only a few training samples. The challenging of few-shot learning is to learn the reliable feature representation of the target. In this study, we use a DCNN for automated representation learning. To overcome the data set constraint, a weak learning is used to boost the overall performance of target detection and classification. The rationale of our approach is that 1) it is impractical to collect a large data set of labeled samples to build a regular machine learning approach for emerging diseases such as NCIP, and 2) few-shot learning could bridge the gap between the large sample size and the limited available knowledge sources for the establishment of reference standard.

One of the challenges in NCIP diagnosis was its different diagnosis with other diseases. In additional of normal cases, in this study, we collected two types of other lung diseases: lung nodule and other pneumonia. We found that it was relatively easy (AUC of 0.95) to distinguish NCIPs from lung nodules except of some cases with large cancers attached to the periphery for our NCIP-Net. We also found that there was significant performance drop in differentiate NCIPs from other pneumonia cases (AUC of 0.83) in comparing to differential diagnosis of lung nodule or normal. After visual examination, we found it was very similar between individual lesions of NCIPs and other pneumonia cases. One of the potential solutions was to analyze the geometrical distributions of the lesions in different patient groups. However, we currently did not have the sufficient samples to form a reliable classification model which will be the research in our future investigation.

So far, machine learning especially deep learning approaches has received a lot of attentions both in academic and in industry. However, the reliability of ML approaches may vary in the same tasks but different datasets with different population bases. In this study, we collected our data from two different data sources to test the reliability of our approach. It was found that the results in two datasets were similar. Although the solution in this study for NCIP diagnosis may not be the optimal, our framework is flexible. Components in our framework can be replaced to improve the specified objective when additional resources such as DCNN model, the number of training samples with labels, and the number of training samples without labels become available. The limitation of this study includes: 1) limited data set for validation; and 2) single country study which may not apply to other racial bases. Both of the limitations are related to the collection of large and variety of data from diversified sources. Our method will serve as the base model for the community to collectively create a ML model to fight this disease. Moreover, the learning framework will be useful in the future similar emergency situation.
